# The efficacy of antibiotic-impregnated calcium sulfate (AICS) in the treatment of infected non-union and fracture-related infection: a systematic review

**DOI:** 10.5194/jbji-8-91-2023

**Published:** 2023-03-22

**Authors:** Connor C. Jacob, Jad H. Daw, Juan Santiago-Torres

**Affiliations:** Department of Orthopaedics, The Ohio State University College of Medicine, Columbus, OH 43201, USA

## Abstract

**Background**: the treatment of infected non-union to allow for bony healing following orthopedic surgery remains a challenge. Antibiotic-impregnated calcium sulfate (AICS) is an effective vehicle for antibiotic delivery, but its efficacy in treating infected non-union in the setting of trauma and fracture remains unclear. **Methods**: this systematic review analyses nine studies from 2002 to 2022 that investigated AICS as a local antibiotic delivery system for cases of fracture-related infection and infected non-union. **Results**: in total, 214 patients who received AICS were included in this review. Of these patients, there were 154 cases of infected non-union or fracture-related infection. Across all studies analyzed, patients treated concurrently with AICS and systemic antibiotics experienced a 92.9 % rate of bony union and a 95.1 % rate of infection eradication. In addition, 100 % of the 13 patients who were treated with AICS alone experienced eradication of infection and successful bony union. **Conclusion**: AICS is an effective method of antibiotic delivery with a low risk profile that results in high rates of bony union and infection eradication even when used in the absence of systemic antibiotics.

## 1 Introduction

Infected non-union presents unique challenges to the surgeon who must eradicate the infection to allow for bony union. The successful treatment of bony defects in the setting of local bacterial infection often requires debridement of infected bone and soft tissue and antibiotic delivery intravenously, orally, or via local delivery (Pincher et al., 2019).

Bone infections are notoriously difficult to treat due to the lack of antibiotic penetrance when delivered via the bloodstream (Lew and Waldvogel, 2004) Alternatively, antibiotics can be impregnated into bone cement and synthetic graft material, such as calcium sulfate and polymethyl methacrylate (PMMA), and be eluted over time at the site of infection (van Vugt et al., 2019; Shi et al., 2022). While systematic review articles by Thahir et al. (2022) and Shi et al. (2022) have recently been published reviewing the efficacy of antibiotic-impregnated calcium sulfate (AICS) in treating osteomyelitis, there is no comprehensive review of the literature exploring its effects in treating infected non-union and fracture-related infection specifically (Shi et al., 2022; Thahir et al., 2022).

In 1892, German physicians were the first to use calcium sulfate as synthetic graft material to address bony defects (Dreesman, 1892). In the past 2 decades however, calcium sulfate has been shown to effectively carry antibiotics, especially vancomycin, tobramycin, and gentamycin, when used as synthetic bone graft (McKee et al., 2002; Gauland, 2011; Boyle et al., 2019).

AICS affords multiple advantages when treating infected non-union in comparison to antibiotic-impregnated PMMA or systemic antibiotics alone. PMMA beads are non-resorbable and require secondary debridement for removal weeks after their initial placement (Li et al., 2009). Calcium sulfate, on the other hand, effectively fills the dead space created by excision of infected tissue while being fully resorbable; therefore, it provides the advantage of requiring only a single procedure without the need to remove the material from the surgical site during a future operation (Wahl et al., 2017). Unlike PMMA, AICS induces bone formation, improves bone remodeling, and increases cartilaginous surface area at the site of the defect, likely via modulation of mesenchymal stem cells (Boyle et al., 2019; Aquino-Martínez et al., 2017; Coetzee, 1980). Furthermore, calcium sulfate is capable of delivering 10 times more antibiotic to the site of infection when compared to antibiotic-impregnated PMMA, and in vitro data indicate that AICS inhibits bacterial growth similarly or more effectively than antibiotic-impregnated PMMA (Wahl et al., 2017; McConoughey et al., 2015).

AICS also has the benefit of reducing the need for high doses of systemic antibiotics. Indeed, a recent investigation by Masrouha et al. (2018) found that AICS alone effectively united bone and eradicated infection without the need for systemic antibiotics (Masrouha et al., 2018). This application of AICS is especially promising for patient populations with poor compliance and in medical settings with limited resources and access to surgical services.

The primary purpose of this systematic review is to investigate the rates of fracture healing and infection eradication of AICS in the setting of infected non-union and similar pathologies. We also discuss complications of AICS noted in the literature and the efficacy of AICS alone in treating these issues sans a prolonged systemic antibiotic regimen.

## 2 Methods

**Figure 1:**
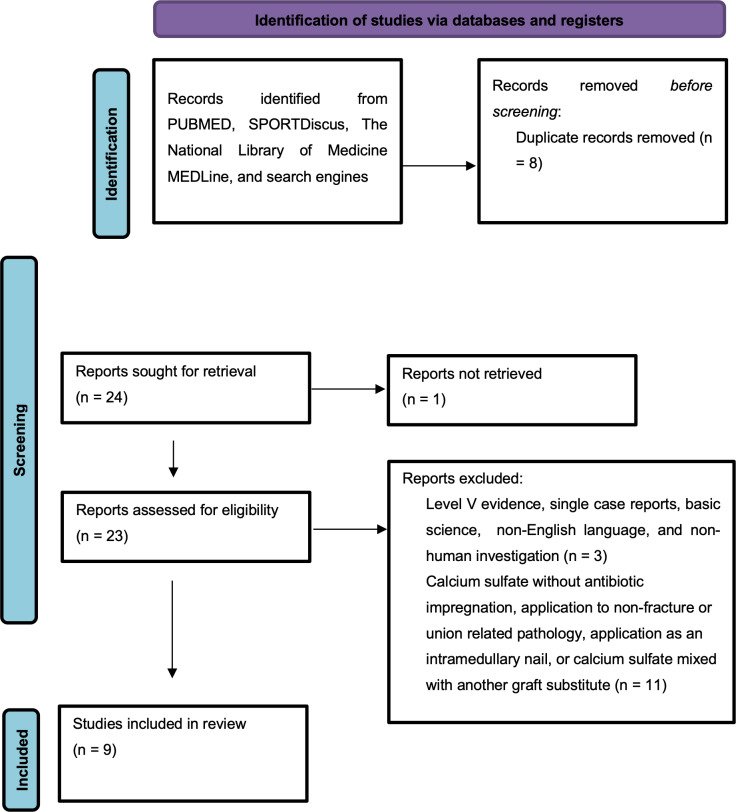
Flow chart describing the systematic review.

A systematic review of the literature was conducted in accordance with the Preferred Reporting Items for Systematic Reviews and Meta-Analyses (PRISMA) checklist (Fig. 1). The search strategy utilized the following search terms: “stimulan” or “calcium sulfate” or “calcium sulphate” and “union.” This search was independently performed by two authors (Connor C. Jacob and Jad H. Daw) on 23 May 2022. The authors utilized the following databases: Pubmed (1950–present), SPORTDiscus (1975–present), and The National Library of Medicine's MEDLine (1964–present). The studies included in this review were classified with evidence levels 1, 2, 3, or 4 according to the Oxford Center for Evidence-Based Medicine. Printed publications and electronically published publications were eligible for inclusion. Web search engines including Google and Bing were used to discover any relevant studies that did not appear in the initial databases.

Our inclusion criteria limited results to English language clinical outcome studies involving the application of antibiotic-impregnated calcium sulfate as a local antibiotic vehicle and bone graft substitute to orthopedic injuries complicated by infection, the potential for infection, infected non-union, or infected bony defect with evidence levels 1–4.

Non-English studies, and studies of level 5 evidence including single-case reports, expert opinions, and personal observation, were excluded. Basic science and animal models investigating the properties of antibiotic elution, antibiotic selection, and mode of calcium sulfate application were excluded. Additionally, studies investigating the use of calcium sulfate alone (without antibiotic) as a bone graft substitute and the application of calcium sulfate with or without antibiotic for purposes other than treating infection and/or non-union of bones were excluded. Studies that investigated the use of antibiotic-impregnated calcium sulfate during the treatment of arthroplasty, hemiarthroplasty, or procedures involving joint prosthesis were also excluded.

Descriptive statistics were calculated with categorical data reported as frequencies (percentages) and continuous data reported as means plus or minus standard deviations from the mean. Any ranges of data were reported as minimum to maximum absolute values. For any and all statistical analyses included within the studies analyzed, p<0.05 was deemed to be significant.

## 3 Results

Nine studies were deemed to be appropriate for inclusion and analysis for this review (Table 1). All the included studies are clinical in nature and investigated AICS used as graft material for infected non-union fracture-related infection and post-traumatic osteomyelitis with bony defect at various surgical sites such as the tibia, femur, humerus, etc. Seven studies were retrospective reviews of patients that received AICS as an adjunct to systemic antibiotic therapy, one study compared AICS to PMMA, and one study investigated AICS as a standalone therapy without the use of a prolonged course of intravenous (IV) or oral antibiotics. The synthetic bone graft material was packed into the bony defect after debridement and excision of infected or necrotic tissue for all patients in each study. The types of calcium sulfate utilized in these studies include the Stimulan Bullet Mat and Introducer from Biocomposites Limited (Patel et al., 2022; Vala and Patel, 2022; Qin et al., 2018; Menon et al., 2018; Masrouha et al., 2018) and OSTEOSET^®^-T from Wright Medical (McKee et al., 2002, 2010; Li et al., 2009; Humm et al., 2014). Stimulan is a resorbable preparation of calcium sulfate commonly used to delivery vancomycin, gentamycin, or tobramycin in bone or soft tissue with setting times ranging from 2–15 min. OSTEOSET^®^-T is a resorbable bone graft material composed of calcium sulfate and 4 % tobramycin that is sold as pellets capable of being packed into bony defects (Noor et al., 2013).

**Table 1:** Results.

Author (year)	Journal	No. of patients (mean age)	Type of AICS	Surgical methods	Pathology	Pertinent outcomes
Cai et al.(2010)	Orthopedics	28 (34.5)	OSTEOSET^®^-T pellets	Internal fixation, IV antibiotics (mean = 2.7 d)	Open fracture of long bones	88.0 % rate of union at 5.8 month mean time to union ( n=28 ), 100 % infection prevention ( n=28 ), 7.6 % rate of complications ( n=28 )
Humm et al.(2014)	Strategies in Trauma and Limb Reconstruction	21 (49)	OSTEOSET^®^-T pellets	External fixation ( n=9 ), soft tissue reconstruction ( n=7 ), oral antibiotics (range = 1.5–6 months)	Post-traumatic osteomyelitis of tibia after fracture	100 % rate of bony union ( n=21 ), 95.2 % rate of infection eradication ( n=21 ), 52 % rate of wound complications ( n=21 ), 1 case of AKI (resolved without intervention in 1 week)
Masrouha et al. (2018)	Strategies in Trauma and Limb Reconstruction	13 (35)	Stimulan	External fixation ( n=13 ), no patient received antibiotics post-discharge	Infected non-union tibia, femur, and humerus	100 % rate of union at mean 5.5 months ( n=13 ), 100 % infection eradication ( n=13 )
Menon et al.(2018)	Journal of Bone and Joint Infection	39 (51)	Stimulan	Oral antibiotics (6 weeks)	Chronic osteomyelitis ( n=25 ), acute osteomyelitis ( n=1 ), periprosthetic joint infection ( n=3 ), soft tissue infection ( n=1 ), and infected non-union ( n=8 )	87.5 % union rate ( n=8 ); 94.9 % infection eradication rate ( n=39 )
McKee et al.(2002) ${}^{a}$	Journal of Orthopedic Trauma	25 (43)	OSTEOSET^®^-T	Internal fixation ( n=5 ), external fixation ( n=12 ), additional autografted bone ( n=4 ), oral antibiotics (mean = 7.2 weeks)	Post-traumatic infected non-union ( n=16 ), bony defects ( n=9 )	87.5 % union rate ( n=16 ) at mean 6.9 months, 92 % infection eradication ( n=25 ), mean defect size 30.5 cm ${}^{3}$
McKee et al.(2010) ${}^{b}$	Journal of Orthopedic Trauma	15 AICS (44.1) 15 PMMA(45.6)	OSTEOSET-T	Internal fixation ( n=6 ), external fixation ( n=9 ), oral antibiotics (mean = 19.2 d, PMMA cohort), oral antibiotics (mean = 20.4 d; AICS cohort)	AICS: infected non-union ( n=8 ), chronic osteomyelitis with bony defect ( n=7 ) PMMA: infected non-union ( n=8 ), chronic osteomyelitis with bony defect ( n=7 )	AICS: 100 % union rate ( n=8 ) at mean 9 months, 86 % infection eradication ( n=14 ) PMMA: 75 % rate of union ( n=8 ), 86 % eradication rate ( n=14 )
Patel et al.(2022) ${}^{c}$	European Journal of Orthopedic Surgery and Traumatology	13 (38)	Stimulan	External fixation ( n=3 ), oral antibiotics (6–12 weeks)	Fracture-related infection of tibia and femur	100 % union at 8 months mean ( n=6 ), 100 % infection eradication ( n=13 )
Qin et al.(2018)	Biomedical Research International	35 (38)	Stimulan	External fixation ( n=35 ), oral antibiotics (minimum 6 weeks)	Post-traumatic and/or iatrogenic fracture-related infection	97.1 % union rate ( n=35 ); 97.1 % infection eradication ( n=35 ), defect mean 9.5 cm
Tamboowalla et al. (2019) ${}^{d}$	Journal of Karnataka Orthopedic Association	25 (39.4)	Stimulan	Internal or external fixation ( n=16 ), parenteral antibiotics in cases of infection (3 weeks), autologous iliac crest bone grafting ( n=16 )	Infected non-union ( n=12 ), aseptic non-union ( n=4 ), osteomyelitis ( n=9 )	87.5 % union rate ( n=16 ), 85.9 % infection eradication ( n=21 ), 92 % return to activity ( n=25 )

${}^{a}$ The rate of bony union includes only the infected non-union cases ( n=16 ), excluding cases of osteomyelitis-related bony defects ( n=9 ). ${}^{b}$ The rate of bony union includes only the infected non-union cases for both the patients who received PMMA ( n=8 ) and those who received AICS ( n=8 ). ${}^{c}$ The rate of bony union includes only the infected non-union cases ( n=6 ), excluding cases of osteomyelitis without non-union ( n=7 ). ${}^{d}$ The rate of infection eradication includes only the infected non-union ( n=12 ) and osteomyelitis ( n=9 ) cases. The rate of bony union includes both the infected and aseptic cases of non-union ( n=16 ). Internal or external fixation, and autologous iliac crest bone grafting, was utilized when deemed necessary by the surgical team for the non-union cases ( n=16 ).

In total, the studies included in this review reported on 229 patients. There was a total of 214 patients who received AICS across all studies and 15 patients who received antibiotic-impregnated PMMA beads in the comparison study by McKee et al. (2010). Of the patients who received AICS, there were 154 cases of infected non-union or fracture-related infection: the 60 other patients were diagnosed with non-fracture-related forms of osteomyelitis. The average age of all patients was 41.3 years. Because this review focuses on the subset of those patients with infected non-union and fracture-related infection, data concerning cases of osteomyelitis not directly related to fracture or non-union are not extensively discussed here: information on the role of AICS in chronic osteomyelitis can be found in other systematic reviews on the topic (Shi et al., 2022; Thahir et al., 2022).

The composite rate of successful union across all patients treated with AICS was 92.9 % at a mean time of 6.4 months and an overall infection eradication rate of 95.1 %. Of the 80 non-union patients who received OSTEOSET^®^-T, 93.3 % achieved bony union at an average time of 6.9 months. The 74 non-union patients who received Stimulan achieved union with a rate of 92.5 % at an average of 5.3 months. While there was no statistically significant difference in union outcomes or infection eradication between the AICS and PMMA groups, the use of AICS was associated with a statistically significant decrease in re-operations compared to PMMA ( p=0.04 ) (McKee et al., 2010). Masrouha et al. (2018) reported a rate of union of 100 % at an average 5.5 months for the 13 patients included in this study (Masrouha et al., 2018). Each of these patients was diagnosed with infected non-union prior to surgery, and none of these patients received systemic antibiotics after leaving the hospital. This stands in contrast to the other eight studies analyzed in this review. Excluding the 13 patients from Masrouha et al. (2018), the remaining 216 patients included in this review received both post-operative IV antibiotics and long courses of oral antibiotics after being discharged from the hospital.

The composite rate of infection eradication across all studies analyzed in this paper was 95.1 %. OSTEOSET-T plays an effective role as an adjunct to systemic antibiotics; indeed, the rate of eradication was 95.2 % with only one patient of the 21 total requiring further debridement to resolve persistent infection (Humm et al., 2014). Of these infections, 38 % were caused by a *Staphylococcus* strain, the most common cause of bone-related infections in the studies analyzed in this review (Humm et al., 2014).

Rates of complications varied between the papers analyzed in this review. Qin et al. (2018) found that pain was the most common complication following surgery (Qin et al., 2018). It was found that the presence of wound leakage ( n=7 ) doubled the risk of wound healing problems in comparison to sites without wound leakage ( n=14 , p=0.06 ) (Humm et al., 2014). Surgeons packed AICS either into the intramedullary cavity or into the defect itself, and no statistical significance was found when comparing rates of wound leakage between these sites ( p=1.0 ). Several studies reported instances of persistent non-union or infection, but these instances were usually isolated or rare, as demonstrated in Table 1. One patient developed an acute kidney infection that resolved without medical or surgical intervention in 1 week (Humm et al., 2014). Furthermore, McKee et al. (2010) found that 7 and 15 re-operation procedures were necessary following the insertion of AICS and PMMA, respectively, into the bony defect (McKee et al., 2010). The article by McKee et al. (2002) includes the fact that sterile draining sinuses ( n=8 ) are fairly common after AICS pellet insertion; however, these resolved upon resorption of the calcium sulfate pellets at an average of 2.7 months (McKee et al., 2002).

## 4 Discussion

While the studies included in this review dealt with a variety of surgical sites, etiologies, and treatment methods, this review focuses on the data relevant to the role of AICS in treating patients with infected non-union and fracture-related infection. It is known that dead space management via graft material such as AICS or PMMA is superior to debridement alone for the remission of infection in bone (Pincher et al., 2019). Unlike systemic antibiotics and antibiotic-impregnated PMMA, the literature has clearly demonstrated that AICS induces bone formation, improves bone remodeling, and increases cartilaginous surface area: these effects are massively important when comparing treatment options for instances of infected non-union especially (Boyle et al., 2019; Aquino-Martínez et al., 2017; Coetzee, 1980). The resorbable nature of AICS provides great utility in the treatment of infected non-unions in comparison to non-resorbable PMMA. AICS application allows patients to avoid secondary procedures necessary to remove non-resorbable material such as PMMA while maintaining similar rates of bony union and infection eradication (McKee et al., 2010). In these ways, AICS provides an excellent platform for addressing cases of infected non-union and fracture-related infection.

AICS is an effective adjunct to systemic antibiotic delivery. However, AICS can also be considered as the sole antibiotic delivery route in the right patient population. The findings of Masrouha et al. (2018) are especially intriguing when considering the utility of AICS in areas of the world with limited surgical infrastructure and in patient populations with poor compliance. It is the first clinical study to demonstrate the efficacy of AICS as the primary antibiotic vehicle for cases of infected non-union: none of the 13 patients received systemic antibiotics after leaving the hospital, yet the rates of infection eradication and of bony union were 100 % at an average time of 5.5 months. This is in comparison to a study by Ferguson et al. (2014) that investigated AICS as an adjunct to systemic antibiotics as a treatment for osteomyelitis: the researchers demonstrated an infection remission rate of 91.8 % (Ferguson et al., 2014). While both studies demonstrated high rates of infection eradication, the results from Masrouha et al. (2018) are compelling. Larger studies are needed to support the potential AICS to stand alone as a vector for antibiotic delivery and to definitively conclude that side effects of systemic antibiotics outweigh the benefits when treating cases of infected non-union and fracture-related infection, especially in the presence of orthopedic hardware.

The potential benefits of the use of AICS outweigh the risks. The most common complication of AICS is wound drainage. This is understood to be the result of sterile effluent draining from the surgical site after AICS insertion (Humm et al., 2014). While AICS has been associated with instances of acute kidney injury, there was only one case of nephrotoxicity which resolved spontaneously within 1 week of diagnosis (Humm et al., 2014). There was also a one case of tibial nerve neuropraxia that required removal of hardware to resolve, but this was likely unrelated to the AICS itself (McKee et al., 2010). Approximately 10 AICS patients experienced refracture. In patients with poor compliance or with poor access, AICS could be appropriate as the sole antibiotic delivery route: as identified by Cheung et al. (2009), intravenous administration of antibiotics is associated with several side effects including infection, stenosis, and thrombosis (Cheung et al., 2009). Antibiotics themselves pose well-known toxicity risks, and AICS provides the opportunity to spare patients from the traditional 6 or more weeks of systemic antibiotics following surgery for infected non-union. However, data on the use of AICS in isolation are very scarce, and the surgeon should only consider this when systemic antibiotic delivery is not a viable option. Further studies would be of great interest.

There are limitations to this review. Firstly, this review is retrospective in nature and lacks standardized control groups. Secondly, there were nine studies including a total of 229 patients that met inclusion criteria. A greater number of patients would increase statistical power and strengthen the applicability of our conclusions. Thirdly, cases of infected non-union and fracture-related infections lack uniformity in etiologic pathogen, location, and lesion size, and variables such as comorbidities, medications, immune status, and prior operations vary widely from patient to patient. These factors have the potential of impacting outcomes. A prospective clinical research study with standardized operative and rehabilitative protocols could mitigate these limitations.

## 5 Conclusion

AICS is effective as a vector for antibiotic delivery and demonstrates efficacy in promoting union of bones after instances of infected non-union and fracture-related infection. The resorbable nature of calcium sulfate can result in sterile drainage, but this risk is outweighed by their efficacy in local antibiotic delivery, use as a synthetic graft, and no need for secondary procedures for removal of beads, as would be the case for PMMA. AICS also offers the potential of efficacious local antibiotic delivery without long-term systemic antibiotic treatment following surgery (Masrouha et al., 2018). These results are especially significant when considering the utility of AICS among patient populations that lack financial resources and easy access to medical and surgical centers.

## Data Availability

The datasets generated during and/or analysed during the current study are available from the corresponding author on reasonable request.
